# Bone-Forming Capabilities of a Newly Developed NanoHA Composite Alloplast Infused with Collagen: A Pilot Study in the Sheep Mandible

**DOI:** 10.1155/2013/296391

**Published:** 2013-10-27

**Authors:** Charles Marin, Ryo Jimbo, Fabio Cesar Lorenzoni, Lukasz Witek, Hellen Teixeira, Estevam Bonfante, Jose Gil, Rodrigo Granato, Nick Tovar, Paulo G. Coelho

**Affiliations:** ^1^Department of Dentistry, UNIGRANRIO, 25071-202 Duque de Caxias, RJ, Brazil; ^2^Department of Prosthodontics, Faculty of Odontology, Malmö University, 205 06 Malmö, Sweden; ^3^Department of Biomaterials and Biomimetics, New York University, New York, NY 10010, USA; ^4^Department of Prosthodontics, Integrated Center for Research, Bauru School of Dentistry, University of São Paulo, 17012-901 Bauru, SP, Brazil; ^5^School of Chemical Engineering, Oklahoma State University, Stillwater, OK 74075, USA; ^6^Postgraduate Program in Dentistry, UNIGRANRIO University-School of Health Sciences, 25071-202 Duque de Caxias, RJ, Brazil; ^7^Department of Periodontology and Implant Dentistry, New York University College of Dentistry, NY 10010, USA

## Abstract

Lateral or vertical bone augmentation has always been a challenge, since the site is exposed to constant pressure from the soft tissue, and blood supply only exists from the donor site. Although, for such clinical cases, onlay grafting with autogenous bone is commonly selected, the invasiveness of the secondary surgical site and the relatively fast resorption rate have been reported as a drawback, which motivated the investigation of alternative approaches. This study evaluated the bone-forming capability of a novel nanoHA alloplast infused with collagen graft material made from biodegradable polylactic acid/polyglycolic acid versus a control graft material with the same synthesized alloplast without the nanoHA component and collagen infiltration. The status of newly formed bone and the resorption of the graft material were evaluated at 6 weeks *in vivo* histologically and three dimensionally by means of 3D microcomputed tomography. The histologic observation showed that newly formed bone ingrowth and internal resorption of the block were observed for the experimental blocks, whereas for the control blocks less bone ingrowth occurred along with lower resorption rate of the block material. The three-dimensional observation indicated that the experimental block maintained the external geometry, but at the same time successfully altered the graft material into bone. It is suggested that the combination of numerous factors contributed to the bone ingrowth and the novel development could be an alternative bone grafting choice.

## 1. Introduction

Oral implant treatment is one of the reliable treatment options in dentistry. Due to the conceptual changes in treatment planning, implants are today placed in a position so that the suprastructure can be reconstructed in both anatomically and aesthetically ideal configuration. However, in cases of severe atrophy especially in the aesthetically demanding maxillary anterior region, bone augmentation to gain volume may be necessary before an implant can be placed to attain suitable bone architecture [1]. Since it has been suggested that bone volume (both height and width) has been considered as an important precondition to achieve long-term functional and aesthetic success [2, 3], a number of surgical techniques using various bone substitutes have been proposed to augment the bone volume [4, 5]. Conversely to particulate graft, which demands extra materials to guarantee space maintenance such as membrane barriers, the onlay graft does not require such approach since it is self-contained and has the potential to support itself by the soft tissue [6]. Additionally, onlay grafts are frequently employed to augment larger bone defects, whereas lamentably to date, the particulate grafting has not much clinical documentation to support its capabilities. 

 Materials used for onlay grafting are similar to those of the particulates, and it is an undeniable fact that some potential drawbacks associated with the use of autogenous [7, 8], allograft, and xenograft [9, 10] materials have been indicated. Although autogenous bone grafting is still the “gold standard,” the procedure is invasive for secondary surgical sites. Moreover, there always exist potential infection risks from allograft and xenograft materials. Further, xenografts have been reported in some long-term clinical studies that they actually interfere with the bone metabolism, and what seems to be maintaining bone volume is just delaying the biological healing [11, 12]. 

Such issues have directed attention towards the development of synthetic bone substitutes, that is, alloplastic [13], which have experienced considerable advances and are anticipated to provide comparable results to those achieved with the autograft [14]. 

In order to achieve requirements in bone tissue engineering, biomaterials, irrespective of their inherent features, should display qualities including osteoconductive and osteoinductive potentials, biocompatible and compatible with native bone in terms of porous and mechanical behavior [15]. Although a large array of alloplastic-based bone substitutes with different chemical and physical features have emerged directed towards successful tissue engineering [9], the tissue response is expected to be different from each other due to inherent characteristics of each material [16, 17]. 

 The physicochemical and topographical aspects of biomaterials play an important role in osteoinductive mechanism in a biomaterial graft [18, 19]. For instance, the release of calcium and phosphate by calcium phosphate-based biomaterial seems to act as the most important factor involved in its bioactivity [20, 21]. In fact, a series of research studies based on the referred material have widely shown its osteoinductive potential [22–27]. Topographically speaking, a synthesized calcium phosphate with a specific microstructure has been reported to enhance the bone metabolism significantly by stimulating the macrophage activities thereby stimulating osteogenesis [19, 28]. Although osteoinduction mechanisms are still essentially unknown [29], it is worth to note that the ultimate aim is developing bioactive bone-graft substitutes suitable to effectively send signals in order to raise levels of osteoprogenitor cells in a physiological manner [6, 30]. Another important aspect is the mechanical properties, since an ideal onlay grafting material should maintain its intended morphology and, however, at the same time be able to be altered to bone. Thus, a graft material that acts as a scaffold and at the same time possessing an excellent mechanical property could fully alter the autogenic bone grafting procedure. 

The aim of this pilot study is to histologically and three dimensionally observe the bone-forming capability of a novel synthetic alloplastic graft material presenting nanoHA and collagen infusion at the nanometer scale aimed for onlay graft application implanted on the bone mandible of sheep and removed at 6 weeks *in vivo*.

## 2. Material and Methods

### 2.1. Synthetic Bone Blocks

**Figure 1:**
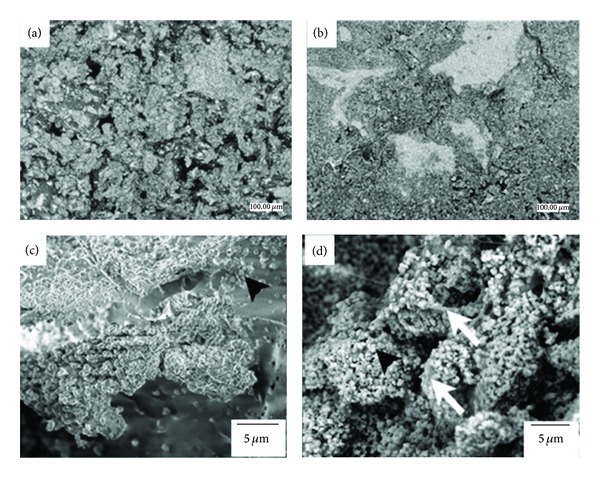
While the macrogeometric structure of both bone blocks were similar. At low magnification, scanning electron micrographs of the difference in HA particle size was easily depicted between (a) control and (b) experimental blocks. Field-emission scanning electron micrographs of the experimental group depicted the (c) nanoHA particles within the biopolymeric matrix (arrowheads) and that (d) collagen infusion took place at the nanometer scale (arrows).

This study utilized two synthetic composite blocks (Intra-Lock International Boca Raton, Florida USA) (Patent Pending) labeled as control and experimental (Figure 1). The two blocks were made of a biocomposite of polylactic acid (PLA)/polyglycolic acid (PGA) and hydroxylapatite particles (HA). The experimental material presented the PLA/PGA scaffold and nanometer scale hydroxylapatite (HA) particles and were infused with collagen (experimental group). The control group presented the PLA/PGA scaffold structure and micrometer scale HA particles. The macrogeometric structure of both bone blocks was similar. At low magnification, scanning electron micrographs the difference in HA particle size was easily depicted between control (Figure 1(a)) and experimental blocks (Figure 1(b)). High magnification field-emission scanning electron micrographs depicted the nanoHA particles within the PLA/PGA matrix (Figure 1(c)) and collagen infusion between nanoHA particles (Figure 1(d)) for the experimental group.

The method of the whole process including the collagen infusion is proprietary and patent pending. The blocks produced for this experiment were cubic, 10 × 10 × 10 mm, and supplied sterile by gamma radiation. The blocks were then shaped with a surgical number 22 blade to a size of 10 mm in height, 10 mm in length, and 5 mm in width to obtain standard sizes for placement.

### 2.2. Animals and Surgery

Four sheep (approximately 6 months of age) were used for the study. This study was approved by the bioethics committee of Ecole Nationale Vétérinaire Maisons-Alfort, Paris, France.

**Figure 2:**
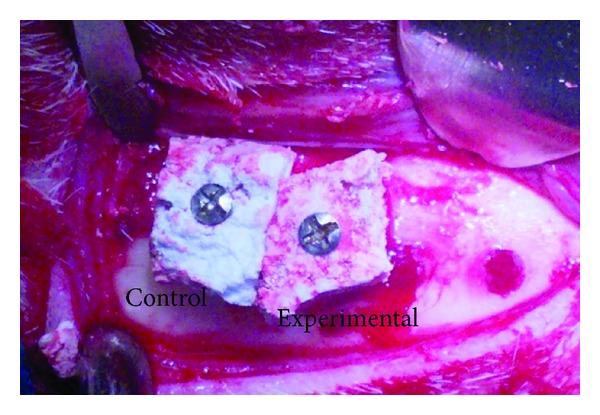
Clinical aspect of extraoral access utilized for the placement of control and experimental blocks. Blood wetting was observed throughout the volume of the experimental block material, whereas blood wetting was lower for the control block.

The central region of the mandibular body on the lateral aspect was chosen for the procedure. All the procedures were performed under general anesthesia. Preanesthesia was made by means of intravenous (IV) Thiopental (15 mg/Kg) followed by orotracheal intubation. The inhalatory general anesthesia was maintained with isofluorane (2.5%), intra-muscular (IM) ketamine (0.2 mg/Kg), and meloxicam (IM, 0.5 mg/Kg). After shaving and exposing the skin, an antiseptic solution with iodine was applied to the surgical site, as well as the surrounding area. An incision of 5 cm was made parallel to the inferior border of mandible. The platysma was dissected and cut in order to reach the periosteum, which was subsequently incised and reflected with periosteal elevator, and, finally, the mandibular body was exposed using manual retractors. The sites were prepared with a straight hand-piece at 900 rpm with abundant saline irrigation, and for each block, 5 perforations of 1.3 mm in diameter were made through the cortical bone. In brief, 4 perforations were made creating an 8 mm square, and an additional perforation was placed in the center of the square for eventual block fixation. Both control and experimental blocks were thereafter placed and fixed with 14 × 1.6 mm screw. After fixation, the stability of blocks as well as the intimate contact among all sides of the blocks to the mandibular body was checked (Figure 2) and appropriate fixation stability was easily achieved due to the block resiliency. After saline irrigation, the surgical sites were sutured layer by layer (internal layers: 3–0 vicryl, skin: 3–0 nylon). Postoperatively, all animals were given antibiotics (benzylpenicillin, 15 mg/Kg and dihydrostreptomycin 20 mg/Kg, IM) for 5 days, analgesics (patch of fentanyl, 3 *μ*g/h/kg, effect during 3 days) on the skin, and anti-inflammatory (meloxicam 0.5 mg/kg, IM) for two days. During the postoperative period, no signs of infection or other complications were observed. Euthanasia was performed after 6 weeks by anesthesia overdose, and the blocks/mandibular body was retrieved. After a careful removal of the surrounding soft tissue, the surgical site was exposed, and stability of blocks was checked. Thereafter, all samples were subjected to histological processing.

### 2.3. Micro-CT Imaging and Histology

 The samples were fixed in 10% phosphate buffered formalin for 24 h and, thereafter, were gradually dehydrated in a series of ethanol concentrations. After dehydration, the samples were infiltrated and embedded in autopolymerizing methyl metacrylate resin. Upon the completion of the curing process, the embedded blocks were scanned by means of microcomputed tomography (*μ*CT 40 Scanco Medical, Brüttisellen, Switzerland). The X-ray energy level was set at 70 kV, and a current of 114 *μ*A, with a slice resolution of 20 *μ*m. All data were exported in DICOM-format and imported in Amira software (Visage Imaging GmbH, Berlin, Germany) for evaluation. Manual segmentation process employing semiautomatic or automatic segmenting tools was used to generate the 3D images. 

 After *μ*CT imaging, all resin-embedded blocks were subjected to undecalcified ground sectioning. One central undecalcified cut and ground section was prepared from each sample with a slow speed precision diamond saw (Isomet 2000, Buehler Ltd., Lake Bluff, USA). The sections were ground to a final thickness of about 90 *μ*m and stained with Stevenel's Blue and Van Giesons Picro-Fuchsin. A slide scanner ScanScope GL (Aperio Technologies, Inc., Vista, CA) was used for the histological observation. 

## 3. Results

 During surgery for block placement, blood wetting was observed throughout the volume of the experimental block material, while noticeable lower wetting was lower for the control block (Figure 2).

Postoperative clinical evaluation revealed that the augmented sites did not present any complication (absence of inflammation, infection, etc.) throughout the 6 weeks healing period. The sheep were allowed to eat as soon as fully recovered from general anesthesia and did not present substantial weight gain or loss thereafter.

 Immediately following euthanasia, sharp dissection of the mandibular region did not reveal any clinical sign of inflammation or infection, and it was clinically evident that no substantial degradation of both biomaterial blocks existed, and those were in the placement position held by the titanium screw. New bone formation was evident in regions surrounding the biomaterial blocks. 

**Figure 3:**
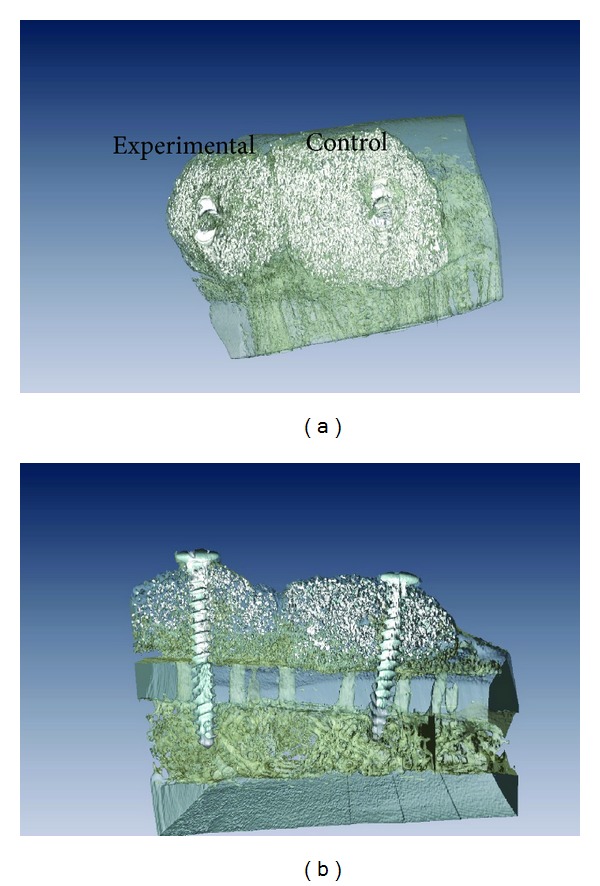
Three-dimensional reconstruction of the mandibular segment containing both experimental and control blocks. (a) Lateral view of the onlays depicted bone ongrowth on the lateral aspects of both blocks. (b) The cross-sectional reconstruction showed the perforations performed in the mandibular lateral aspect cortical (arrows). Bone ingrowth was observed at the region in immediate contact with the mandibular bone for both blocks; bone ingrowth throughout the volume of the block was only observed for the experimental group, which also presented lower amounts of synthetic material compared to the control group.

 The *μ*CT reconstruction of the augmented regions showed bone growth around both control and experimental blocks (Figure 3). Bone ongrowth onto the biomaterial block surfaces was depicted for both groups. However, while bone ingrowth was observed at the region in immediate contact with the mandibular bone for both blocks, bone ingrowth throughout the volume of the block was only observed for the experimental group, which also presented lower amounts of synthetic material compared to the control group (Figure 3). 

**Figure 4:**
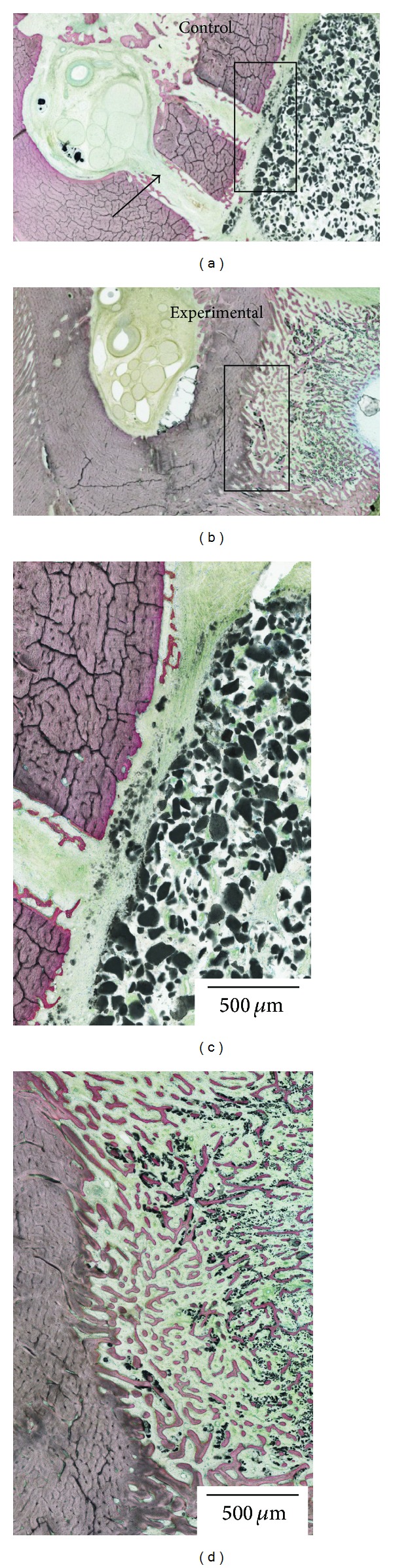
Histologic sections for the (a) control and (b) experimental block materials placed on the lateral aspect of the mandible. The histologic sections revealed the perforations performed in the mandibular lateral aspect cortical (arrows). Higher magnification of the (c) control and (d) experimental blocks showed that bone ingrowth occurred throughout the volume of the experimental block material, and little ingrowth occurred for the control block material. Smaller amounts of synthetic material were also observed for the experimental block relative to the control block material.

The histologic sections confirmed the difference in healing pattern observed through three-dimensional reconstruction (Figure 4), where bone ingrowth occurred throughout the volume of the experimental block material, and little ingrowth occurred for the control block material. Smaller amounts of synthetic material were also observed for the experimental block relative to the control block material.

## 4. Discussion

It has been a general consensus that the expected clinical outcome of ectopic onlay grafts intended for lateral or vertical augmentation is unstable regardless of the graft origin. This is due to the fact that initial vascularization (blood supply) occurs only from the bottom of the graft and may be insufficient for the graft to be successfully altered by newly formed bone before the graft collapses from the constant tension of the soft tissue or is resorbed by the active macrophages. 

The histological and three-dimensional observation of the control sites depicted that the graft material lacked new bone ingrowth and for some locations, and soft tissue encapsulation could be observed. The control alloplast at 6 weeks presented some degree of soft tissue incorporation inside the block. On the other hand, active new bone ingrowth was notable within the experimental graft material. Of note is that compared to the control blocks where the block maintained its shape both externally and internally, the experimental blocks seemed to have degraded and/or resorbed, while newly formed bone filled the spaces originally occupied by the grafting material bulk. Further, remaining block material seemed to be in contact to the aligning newly formed bone. This is an indication that bone metabolism has been stimulated due to the graft material and is in accordance with the reports from Ono et al. (2011), where in their lateral augmentation model, they found more amount of newly formed bone for surface modified beta-tricalcium phosphate blocks than the control without modification [19]. By using enzyme histochemistry, they confirmed that vigorous osteoclastic activity accompanied new bone formation expressing alkaline phosphatase, presenting constant block material resorption and new bone apposition simultaneously. It was suggested that successful bone alteration could be dependent on the topography (including porosity) and chemistry of the synthesized blocks as in the case for the present pilot study. Since the major difference in the two synthesized blocks is the HA particle size and collagen infusion, such nanometer scale features may be separate or in tandem be accounted for the difference in bone healing kinetics. 

He et al. (2012) have previously reported that the infiltration of collagen to porous hydroxyapatite improved mesenchymal stem cell adhesion, proliferation, and differentiation [31]. They suggested that the self-reconstruction property of the collagen enhanced fibrous network formation, and it can be a potential carrier for other proteins and cytokines. This effect has been clinically suggested to be effective as Simion et al. have shown that collagen matrix could be applied for an effective scaffold for tissue regeneration [32]. 

Another added feature by the thorough infiltration of collagen is increased hydrophilicity. Our surgical procedures showed that immediately after placement of the experimental blocks to the decortified surgical site, blood infiltrated the entire block indicating its extreme hydrophilicity compared to the control blocks. This has been reported to be one of the features when collagen is combined with biodegradable materials that the collagen increases the material hydrophilicity [33]. As it has been well described that the hydrophilicity is important for osteogenic cell responses [34–37], it can be assumed that the physiological cascade of events further leads to the formation of new bone. 

Finally, it can be speculated that the improved bone-forming properties seen with the experimental block were achieved due to the combination of multiple factors such as the structural biomechanical strength that maintained the original external geometry, the micrometer level and related nanometer scale structure of the composite created by the synthesis and infiltration of the collagen and its distribution, surface energy including hydrophilicity, and perhaps others. Multivariable experimental studies are under way to explore the potential of this promising novel grafting material.
